# Short Implants and Indirect Sinus Lift Versus Direct Sinus Lift With Standard-Length Implants: A Case Report

**DOI:** 10.7759/cureus.65197

**Published:** 2024-07-23

**Authors:** Rachel Changrani, Amod Patankar, Swapna A Patankar, Pranjali Kulkarni, Amisha Sharma

**Affiliations:** 1 Oral and Maxillofacial Surgery, Bharati Vidyapeeth Dental College and Hospital, Pune, IND; 2 Oral Pathology and Microbiology, Bharati Vidyapeeth Dental College and Hospital, Pune, IND

**Keywords:** sinus augmentation, direct sinus lift, indirect sinus lift, short implants, sinus lift procedure

## Abstract

The choice of implant length in relation to the available bone quality and quantity and biting force is a critical factor in the success of implants and longevity of the prosthesis. Long implants have always been considered more desirable in this respect but in patients with advanced alveolar bone resorption, their placement is problematic due to the anatomic boundaries. This holds more true in relation to the posterior maxilla wherein the residual crestal bone height is usually compromised due to pneumatizing sinus floor.

In this study, we have incorporated the use of short implants in conjunction with indirect sinus lift for cases with severely resorbed posterior maxillary edentulous regions to avoid direct sinus lift surgery and increase patient comfort. A 63-year-old patient had tooth 16 missing and wanted implant rehabilitation. The residual alveolar bone height was 3 mm. Short implant placement after indirect sinus lift was achieved with good primary stability. Prosthetic loading was performed after six months. One year follow-up showed no complaints or discomfort.

In cases where the residual alveolar bone height of the edentulous space in the posterior maxilla was less than 4 mm, the use of indirect sinus lift with placement of short implants (height < 6 mm) proved to be advantageous over a direct sinus lift procedure with delayed placement of standard-length implants. This technique was less time-consuming, less surgically morbid, and more economical. The patient compliance was superior and no complaints were faced in a one-year follow-up period.

## Introduction

The posterior maxilla in long-standing edentulous cases often offers great difficulty in implant rehabilitation due to the anatomic limitations, mainly the pneumatized sinus floor. Such cases often require sinus floor grafting to be able to place standard-length implants. These techniques are sensitive, costly, time-consuming, and increase surgical morbidity [1].

Short implants have widely gained popularity in the world of implant dentistry as their use avoids the need for patients to undergo complex surgical procedures for ridge augmentation and acquire adequate height for standard-length conventional implants, with not much difference in the success rates. A cohort study conducted by Lorenz et al., which aimed to evaluate whether a reduced implant length had any impact on implant success and peri-implant hard and soft tissue health in implants placed in the posterior maxilla to avoid sinus augmentation procedures, showed a 100% success rate after a follow-up period of five years. The implants did not show any signs of peri-implant infections and the study concluded that “short implants” are a reliable treatment option to avoid sinus augmentation procedures for implant placement [2].

The lateral approach of sinus augmentation or the direct sinus lift procedure as described by Tatum in 1977 has been used as a treatment modality for severely pneumatized sinus and less than 4 mm of residual crestal bone height for many years. Though this technique laid a foundation for modern sinus augmentation for implant rehabilitation, the technique is very sensitive and may lead to many surgical complications [3].

This technique of placing short implants after performing a crestal approach or indirect sinus lift bypasses the need for performing a direct sinus lift technique, thus preventing the need to perform a highly technique-sensitive procedure and decreasing surgical morbidity.

## Case presentation

Misch classified the residual crestal bone height below the sinus floor based on the dimension and the required procedure for sinus augmentation [4]. According to this classification, SA-1 is when the sub-antral bone height is more than 10 mm. Such cases do not require any sinus augmentation procedure. SA-2 is when the sub-antral height is 8 mm in which an indirect sinus lift procedure is indicated. SA-3 are cases where the height is 5-8 mm, wherein indirect sinus lift using bone graft or direct sinus lift with/without the placement of an implant in the same sitting may be performed. SA-4 are cases in which the bone height is <5 mm and such cases require a direct sinus augmentation procedure with delayed placement of an implant.

Here, the authors recommend using short implants with a height < 6 mm for implant rehabilitation in the posterior maxilla with severely pneumatized sinus. This eliminates the need for extensive sinus floor augmentation. Thus, in this scenario, SA-3 and SA-4 cases may be treated by indirect sinus lift followed by implant placement with/without the use of bone graft.

An indirect sinus lift may be performed using various techniques such as osseodensification, the use of osteotomes, and the hydraulic lift technique, and a short implant should be placed in the prepared osteotomy site. Bone grafts may be used based on the requirements of the particular case. The implanted site should be allowed time for osseointegration for four to six months. Delayed loading of implant prosthesis should be preferred [3].

**Figure 1:**
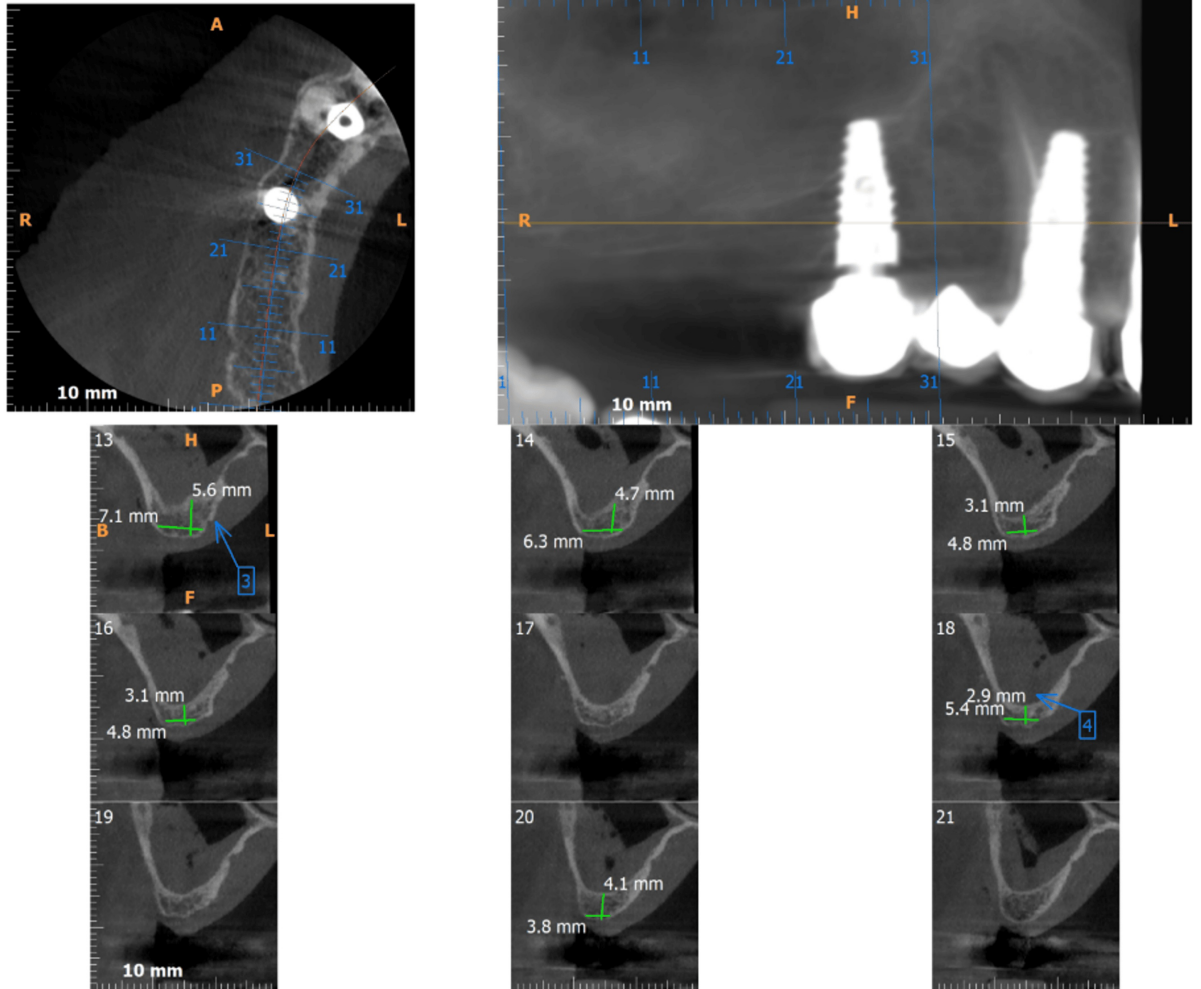
Preoperative cone beam computed tomography showing reduced residual alveolar bone height in region 16.

A 63-year-old male patient had come to our institute with atrophic posterior edentulous maxilla with missing teeth 16 and 17. He underwent a preoperative cone beam computed tomography (CBCT) that revealed residual crestal bone height in region 16 to be 2.9-3.1 mm (Figure 1). He was advised a treatment plan of direct sinus lift augmentation followed by standard-length implant placement and delayed loading of the prosthesis.

**Figure 2:**
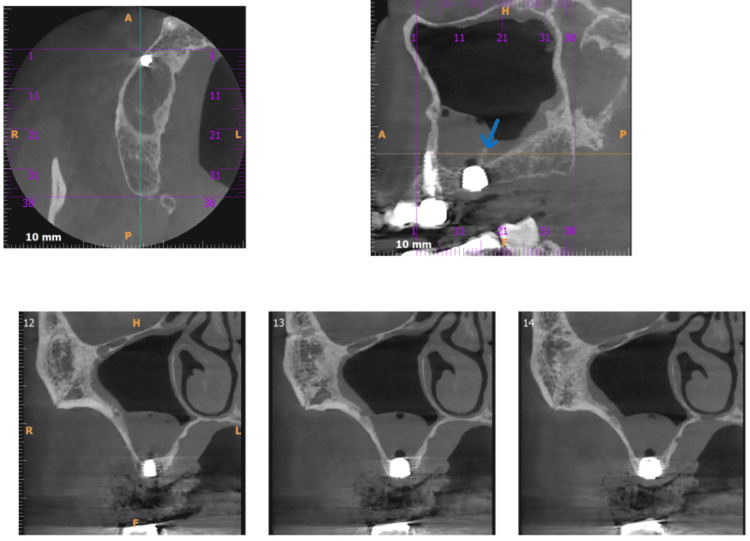
Immediate postoperative cone beam computed tomography after indirect sinus lift and short implant placement. The arrow shows blood pooling over the implant and bony fragments from the sinus lift procedure.

**Figure 3:**
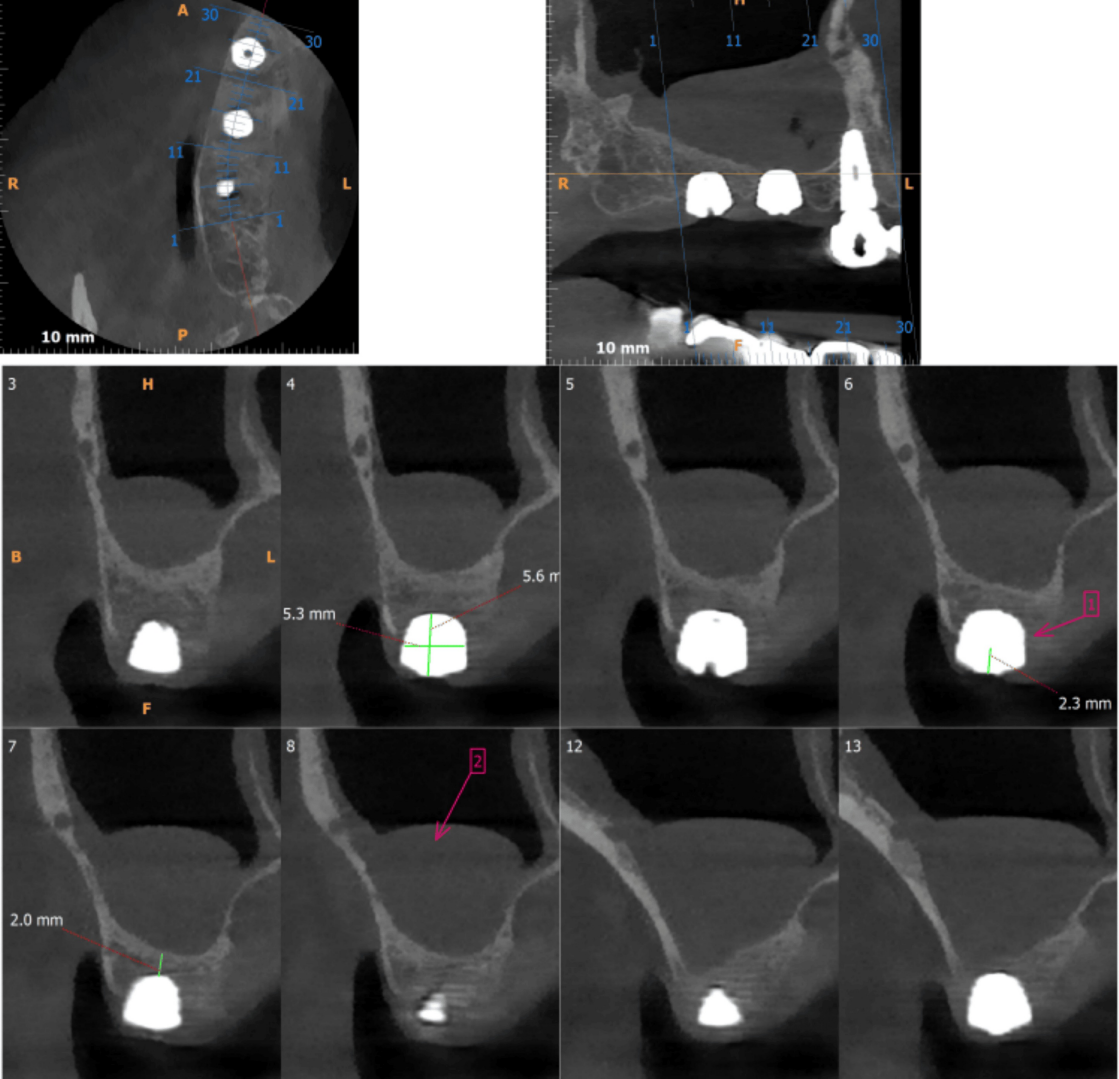
Six-month postoperative cone beam computed tomography showing the osseointegrated implant in region 16 with sound bone surrounding the implant.

The patient refused the proposed treatment plan as he found it to be very time-consuming. An alternate plan was proposed, which was indirect sinus augmentation with placement of short implants, followed by delayed loading of prosthesis. All the information about the technique was explained and informed consent was obtained. The patient gave a history of chronic sinusitis and was informed about the risks of implant failure and other related complications. The procedure was performed under local anesthesia. Indirect sinus lift was performed using osteotomes and a 5.2 * 6 mm implant was placed in region 16. An immediate CBCT was performed after the procedure, which showed bony spicules in the maxillary sinus over the implant (Figure 2). Another CBCT was obtained after six months of the procedure before loading the implant, which showed successful osseointegration of the implant with sound bone surrounding the implant (Figure 3). A one-year follow-up after delivering the final prosthesis was obtained wherein no mobility or bone loss was observed.

## Discussion

The discovery of the direct sinus lift procedure by Tatum in 1977 was a game-changing point for implant-supported rehabilitation in posterior maxillary edentulous spaces. These procedures allowed implantologists to be able to place conventional implants in severely resorbed alveolar ridges [5].

Later, Tatum described the method for indirect sinus lift in 1986, which was further popularized by Summers in 1994 [6]. This gained a lot of popularity since it was less invasive and did not require a need for a lateral window. This technique also portrayed a shorter list of complications as compared to the direct sinus lift technique, but it offered a limited increase in bone height [6] and cannot be used in SA-3 and SA-4 cases for placement of standard-length implants.

The use of short implants has started to gain recognition as they eliminate or reduce the need to perform a vertical ridge augmentation procedure in both atrophic posterior maxilla and posterior mandible cases. Many studies have been published that demonstrate the success of short implants in such cases. A systemic review by Chen et al. showed no difference in the survival rates of short implants and long implants [3,7]. In cases with atrophic posterior maxillary edentulism, many studies have been conducted that conclude that short implants can be a successful alternative to direct sinus lift procedures [3,6].

Combining the technique of indirect sinus lift, which reduces the complications of the surgery as compared to a direct sinus augmentation, with the advantages of using shorter implants, gives us a comparable treatment outcome with lesser risks, reduced cost, and increased patient comfort [8-12]. This technique may be used in patients who contraindicate invasive surgical procedures or those who are unwilling to undergo a direct sinus augmentation procedure.

## Conclusions

The proposed technique of indirect sinus lift with the placement of short implants to avoid direct sinus augmentation could be a resourceful technique that is less technique-sensitive and less time-consuming. It also shows greater patient compliance and satisfaction. With advances in sinus augmentation procedures and the success of short implants, the surgical morbidity associated with direct sinus augmentation procedures can be eliminated.
